# Hearing Loss and Irritability Reporting Without Vestibular Differences in Explosive Breaching Professionals

**DOI:** 10.3389/fneur.2020.588377

**Published:** 2020-12-16

**Authors:** Claire M. Modica, Brian R. Johnson, Christopher Zalewski, Kelly King, Carmen Brewer, John E. King, Angela M. Yarnell, Matthew L. LoPresti, Peter B. Walker, Kristine C. Dell, Elena Polejaeva, Alycia Quick, Bobby Arnold, Eric M. Wassermann, James R. Stone, Stephen T. Ahlers, Walter Carr

**Affiliations:** ^1^Naval Medical Research Center, Silver Spring, MD, United States; ^2^Walter Reed Army Institute of Research, Silver Spring, MD, United States; ^3^Audiology Unit, Otolaryngology Branch, National Institute on Deafness and Other Communication Disorders, National Institutes of Health, Bethesda, MD, United States; ^4^Independent Researcher, Bethesda, MD, United States; ^5^Military Emergency Medicine Department, Uniformed Services University, Bethesda, MD, United States; ^6^DoD Joint Artificial Intelligence Center, Washington, DC, United States; ^7^Behavioral Neurology Unit, National Institutes of Health, National Institute of Neurological Disorders and Stroke, Bethesda, MD, United States; ^8^Department of Psychology, The Pennsylvania State University, University Park, State College, PA, United States; ^9^Department of Clinical and Health Psychology, University of Florida, Gainesville, FL, United States; ^10^School of Psychology, University of Glasgow, Glasgow, United Kingdom; ^11^The Henry M. Jackson Foundation for the Advancement of Military Medicine Inc., Bethesda, MD, United States; ^12^Department of Radiology and Medical Imaging, University of Virginia, Charlottesville, VA, United States; ^13^Oak Ridge Research Institute for Science and Education, Oak Ridge, TN, United States

**Keywords:** hearing loss, blast overpressure, symptom reporting, career breaching, vestibular

## Abstract

**Background:** Blast exposure is a potential hazard in modern military operations and training, especially for some military occupations. Helmets, peripheral armor, hearing protection, and eye protection worn by military personnel provide some acute protection from blast effects but may not fully protect personnel against cumulative effects of repeated blast overpressure waves experienced over a career. The current study aimed to characterize the long-term outcomes of repeated exposure to primary blast overpressure in experienced career operators with an emphasis on the assessment of hearing and vestibular outcomes.

**Methods:** Participants included experienced “breachers” (military and law enforcement explosives professionals who gain entry into structures through controlled detonation of charges) and similarly aged and experienced “non-breachers” (non-breaching military and law enforcement personnel). Responses to a clinical interview and performance on audiological and vestibular testing were compared.

**Results:** Hearing loss, ringing in the ears, irritability, and sensitivity to light or noise were more common among breachers than non-breachers. Breachers reported more combat exposure than non-breachers, and subsequently, memory loss and difficulty concentrating were associated with both breaching and combat exposure. Vestibular and ocular motor outcomes were not different between breachers and non-breachers.

**Conclusion:** Hearing-related, irritability, and sensitivity outcomes are associated with a career in breaching. Future studies examining long-term effects of blast exposure should take measures to control for combat exposure.

## Introduction

In operations and training, U.S. military personnel are exposed to blast overpressure and associated sound pressure insults. These exposures arise from multiple sources, to include improvised explosive devices, ordnance (breach explosives, hand grenades), and weaponry (heavy shoulder-fired weapons, high-powered rifles). It can be difficult to control circumstances surrounding operational assaults, but training environments, on the other hand, typically employ measures to prevent exposures from ordnance or weaponry that may result in an injury. However, there is a growing concern regarding the long-term effects of cumulative subconcussive blast events. Recently, the U.S. Congress directed the Department of Defense to study the effects of blast on military personnel (1, 2). There is particular concern over outcomes from repetitive subconcussive low-intensity blast exposure where symptoms may manifest over time (3).

“Breachers” are military and law enforcement personnel who use controlled explosive breaching charges to quickly ingress into a fortified structure. Some breachers report deleterious effects which they believe are associated with their exposure and coined the term “breacher's brain” to describe a symptom array that includes fatigue, thinking difficulty, and headache (4). Despite this awareness, the effects of blast on military personnel have been difficult to characterize. Assessments of breachers have been inconsistent in detecting changes in neuroimaging, symptom-reporting, neurocognitive performance, or biomarkers of brain injury (5–7).

More recently, LaValle et al. found that exposure to high blast overpressure in breaching training (above 34.47 kPa) led to a transient, but measurable, effect on neurocognitive performance (8). In another recent study examining relatively lower blast overpressure readings at a grenade range training course (0.97–2.89 kPa), Sajja et al. found that post-training neurosensory symptoms were associated with low-level sound overpressure exposure (9). Specifically, headache and thinking difficulty, followed by lightheadedness, ringing of the ears (i.e., tinnitus), restlessness, frustration, and irritability. The authors noted that sound pressure was likely influential in generating the symptoms, as acoustic sensors in the field recorded sound pressure ranging from 153.72 to 163.22 dB peak (9). These readings were in excess of the Department of Defense 2015 Regulation (No. 385-1-89) impulse noise safety limit of 140 dB peak (10). This suggests that sound pressure may be contributing to observed long-term outcomes associated with blast exposure.

The vestibular system may be affected when the auditory system is transducing high-decibel sound waves, as dizziness or imbalance are symptoms commonly reported after blast. In a review of studies examining vestibular metrics, up to half of blast-exposed individuals exhibit vestibular, balance, or ocular motor dysfunction (11). Our goal was to investigate whether career breachers demonstrated auditory or vestibular system dysfunction, not from acute blast exposure or a recent concussion, but from cumulative controlled low-level blast exposure during the course of their career. The data presented here are a component of a large multi-institutional effort to assess the effects of repetitive blast exposure in experienced breachers.

## Methods

### Participants

This study was approved by Institutional Review Boards (IRB) at the Naval Medical Research Center (NMRC.2011.0002), the Walter Reed Army Institute of Research (WRAIR #1796), and the Central Nervous System IRB of the National Institutes of Health. Participants traveled to Bethesda, Maryland for several days of data collection at the National Institutes of Health Clinical Center. Methods on neuroimaging, blood component, and neuropsychological data collected from this study, as well as neuroimaging analysis, have been described in a publication authored by Stone et al. (12). Future publications or reports will focus on analysis of blood, neuropsychological, and posture/balance data collected in this study. Participants were recruited from military and law enforcement communities (active duty and prior service). Breachers (n = 20), were categorized as individuals with careers utilizing explosives to gain entry into structures. Specifically, breachers met the inclusion criteria of at least 4 years of experience with breaching operations occurring at least annually or had exposure to at least 400 breaching blasts over their careers. Non-breachers (*n* = 14) were recruited to match a similar age, education, and years in career to the breachers. Thus, inclusion criteria for non-breachers consisted of at least 4 years of military or law enforcement experience, and exposed to no more than 40 breaching blasts over their careers. All participants were male due to breaching careers only recently opening up to female service members. Exclusion criteria included history of diagnosis of moderate to severe brain injury, central nervous system disorder, medical conditions affecting cerebral metabolism, recent concussion, or injury including loss of consciousness >5 min.

### Clinical Interview

Participants underwent a demographic and historical clinical interview and a medical history and neurological examination. Clinical interview questions and summary responses are detailed in the Appendix (Supplementary Material) and Supplementary Table 1. Responses were recorded and whenever possible, questions were coded into “yes/no” responses. In addition to demographics, participants were asked about their past weapons use and combat experiences. Participants were asked to recount each large weapon or explosive they had interacted with and the number of times they were exposed to its detonation. Responses regarding small arms were not consistently detailed or discussed during interviews, so any responses which were recorded were excluded from analysis. Weapons were grouped into categories of heavy weapons, artillery, small explosives, and large explosives (excluding breaching charges). If a participant reported more than 10 instances of any of the categorized weapons, they were scored as having exposure to that category.

For combat experiences, participants were administered a 34-item Combat Exposure Checklist, a modification of the Walter Reed Army Institute of Research Combat Experiences Scale [described by Guyker et al. (13)] (grammatical tense was adjusted to account for all deployments, three questions related to crime were removed, four questions associated with recent conflicts were added, and answer choices were adjusted). The list contained experiences common to modern battlefields. Participants responded on an ordered scale: experience had never occurred (1), occurred once (2), occurred between two and four times (3), or occurred ten or more times (4). These responses were scored as: 0 = zero experiences; 1 = one experience; 3 = two through four experiences; 10 = 10 or more experiences. Scores for each participant were summed and treated as a scalar variable in the range of 0–340.

### Audiometric, Vestibular, and Balance Data Collection

Audiology and vestibular data were collected and processed in the Audiology Unit at the National Institute on Deafness and Other Communication Disorders. All audiologic evaluations were conducted using GSI-61 (Grason-Stadler, Eden Prairie, MN) clinical audiometers with the patient in double-walled sound treated rooms, both of which met American National Standards Institute criteria (14, 15). Audiological measurements included speech reception thresholds and pure-tone air conduction thresholds measured in octave band frequencies from 250 to 8,000 Hz and including interoctave assessment at 3,000 and 6,000 Hz; bone conduction pure-tone thresholds from 250 to 4,000 Hz were evaluated when air-conduction thresholds exceeded 25 dB HL. Tympanograms were acquired using a Grason-Stadler Tympstar immittance bridge in response to a standard 226-Hz probe tone.

Vestibular testing included measurement of the vestibulo-ocular reflex elicited by stimulation of the horizontal semicircular canal during bi-thermal caloric irrigations and sinusoidal harmonic acceleration using a rotary chair. Sinusoidal harmonic acceleration stimuli were presented using a calibrated Neuro Kinetics (Neuro Kinetics, Inc.; Pittsburgh, PA) Neuro-Otologic Test Center via VEST™ software at octave frequencies from 0.01 to 0.64 Hz, and bithermal air caloric irrigations were delivered via an ICS Medical Chartr NCA-200 irrigator. Ocular motor stimuli were presented in the NOTC light-proof enclosure (Neuro Kinetics, Inc.; Pittsburgh, PA). Eye tracking for all assessments were measured with Neuro Kinetics (Neuro Kinetics, Inc.; Pittsburgh, PA) binocular infrared digital 250-Hz video-goggles via I-Portal-VOG® software.

Cervical vestibular evoked myogenic potentials (cVEMPs) were elicited via an air-conducted 500 Hz tone burst (Blackman gating, 2 ms rise/fall time, 0 ms plateau) presented monaurally via insert earphones at 100–107 dB nHL and a rate of 5.1/s (Intelligent Hearing Systems; Miami, FL). Myogenic activity was recorded from surface electrodes placed on the ipsilateral sternocleidomastoid muscle (reference), the sternum (active), and the forehead (ground). Cervical VEMP responses were accepted only when sternocleidomastoid myogenic activity was between 50 and 100 μV. The cVEMP was interpreted based on presence or absence of the bi-phasic P1-N1 peak response and interaural symmetry ratio of the P1-N1 amplitude.

### Statistics

To compare demographic information and responses to clinical interview questions between breachers and non-breachers, categorical variables were compared by Chi square test, ranked variables were compared by Mann Whitney *U*-test, and scalar variables were compared by one-way ANOVA. Answers to clinical interview questions varying between groups were probed for associations with combat exposure: combat exposure scores were compared by ANOVA between participants reporting “yes” vs. those reporting “no.” After data collection, one breacher participant was discovered to have a vestibular schwannoma; none of his audiological or vestibular data were analyzed as a result. His self-reported clinical interview responses were included, as exclusion of it did not change results. Audiometric and vestibular data were compared by one-way or two-way ANOVA between groups with Bonferroni post-test, depending on whether the test had a single measurement or multiple measurements taken, respectively. Pure tone assessment was analyzed by repeated measures two-way ANOVA as each frequency is dependent on the others; standardized residuals for each ear were compared by one-way ANOVA to compare individual frequencies.

## Results

Race, ethnicity, marital status, handedness [*p* > 0.05: $\chi_{\left(4,\text{N}=34\right)}^{2}$ = 2.893; $\chi_{\left(1,\text{N}=34\right)}^{2}$ = 0.068; χ^2^(3, *N* = 34) = 0.971; $\chi_{\left(1,\text{N}=34\right)}^{2}$ = 0.146], age, years of education, and years of service [*p* > 0.05: *F*_(1,32)_ = 0.078; *F*_(1,32)_ = 0.058; *F*_(1,32)_ = 1.463] were not different between breachers and non-breachers. It should be noted that most participants in either group were right-handed (18 out of 20 breachers and 12 out of 14 non-breachers). Breachers were a mean of 39.7 ± 8.3 years of age (ranging age 26–54 years) and served for 16.8 ± 6.7 years while non-breachers were 38.9 ± 7.8 years of age (ranging 27–53 years) and served for 13.9 ± 7.0 years. Unexpectedly, breachers reported having more head injuries in comparison to non-breachers [1.1 ± 1.0 to 0.3 ± 0.5, *F*_(1,32)_ = 7.712, *p* = 0.009]. In terms of breaching experience, total years exposed to breaches averaged 14.4 ± 7.6 in breachers in comparison to 0.04 ± 0.13 years in non-breachers [*F*_(1,32)_ = 50.323, *p* < 0.001]. Approximate number of total breaches experienced ranged from 100 to 34,800 among breachers, but only 0-15 among non-breachers (*U* = 0, *p* < 0.001).

In terms of other service experiences, breachers were more likely than non-breachers to have exposure to large explosives (besides breaches) [$\chi_{\left(1,\text{N}=34\right)}^{2}$ = 4.568, *p* = 0.033], but no differences were detected among exposure to small explosives, artillery, or heavy weapons. In addition, breachers reported significantly higher combat exposure scores than non-breachers [158.6 ± 65.1 to 60.4 ± 50.0, *F*_(1,32)_ =22.526, *p* < 0.001]. To address the potential confounding of combat exposure in our comparisons, we attempted to compare only those breachers with similar combat exposure scores to non-breachers by limiting the combat exposure score to 160, the highest non-breacher score. However, even when comparing this sub-group of 11 breachers with all 14 non-breachers, the average combat exposure score among breachers was still higher when compared to non-breachers [109.0 ± 25.5 to 60.4 ± 50.0, *F*_(1,23)_ = 8.597, *p* = 0.007].

**Figure 1:**
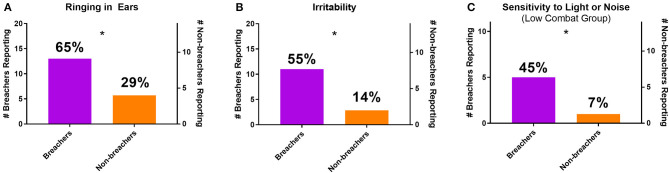
Number and percentage of breachers and non-breachers reporting **(A)** ringing in the ears, **(B)** irritability, and **(C)** sensitivity to light or noise (including only participants with combat exposure scores of 160 or less). *p < 0.05, χ^2^.

From the clinical interview, self-report measures that did not associate with combat exposure were compared between groups. More breachers reported experiencing tinnitus [$\chi_{\left(1,\text{N}=34\right)}^{2}$ = 4.371, *p* = 0.037] and irritability [$\chi_{\left(1,\text{N}=34\right)}^{2}$ = 5.781, *p* = 0.016] than non-breachers (Figures 1A,B). When comparing only the breachers with a matching range of combat exposure scores to non-breachers, low combat breachers reported more sensitivity to light and noise [χ^2^(1, *N* = 25) = 4.957, *p* = 0.026, Figure 1C]. Memory problems [$\chi_{\left(1,\text{N}=34\right)}^{2}$ = 4.371, *p* = 0.037] and difficulty concentrating [$\chi_{\left(1,\text{N}=34\right)}^{2}$ = 5.781, *p* = 0.016] were reported more among all breachers, but these responses were also associated with higher combat exposure scores. The combat exposure score was significantly higher among breachers reporting memory problems [189.3 ± 59.2 to 101.6 ± 23.6, *F*_(1,18)_ = 13.883, *p* = 0.002], and among breachers reporting difficulty concentrating [187.9 ± 64.8 to 122.8 ± 46.7, *F*_(1,18)_ = 6.359, *p* = 0.021]. In contrast, these associations were not seen in breachers reporting ringing in the ears [173.5 ± 67.8 to 131.0 ± 53.3, *F*_(1,18)_ = 2.044, *p* = 0.170], irritability [170.5 ± 64.0 to 144.1 ± 67.1, *F*_(1,18)_ = 0.803, *p* = 0.382], or sensitivity to light and noise [115.0 ± 29.5 to 104.0 ± 23.3, *F*_(1,9)_ = 0.480, *p* = 0.506]. Additionally, breachers reported exercising more hours per week than non-breachers (*U* = 77.500, *p* = *0.028*), but this effect was driven by three breachers reporting 14–30 h per week (all other participants reported 12 or less), so activities which qualify as exercise may have been interpreted differently among participants. No other responses from the clinical interview were statistically significant between groups (*p* > 0.05, χ^2^ or *U*; responses summarized in Supplementary Table 1).

**Figure 2:**
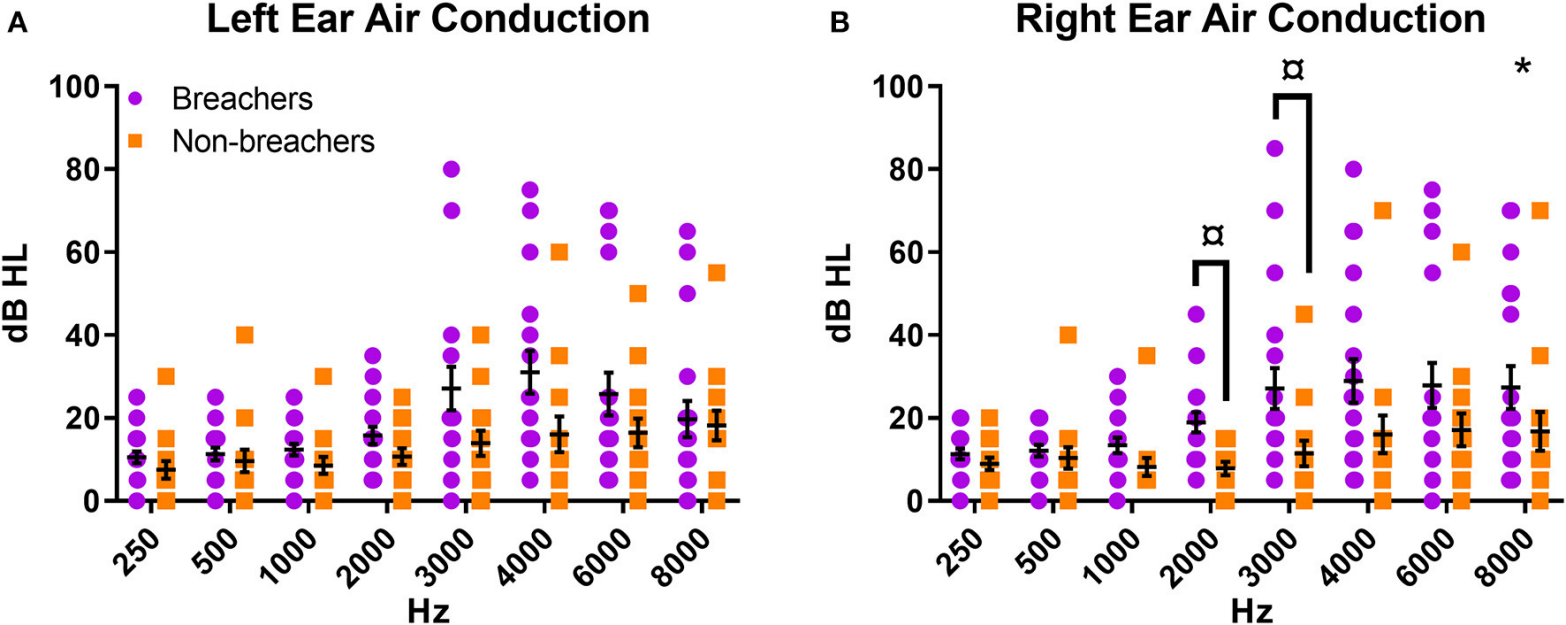
Air conduction pure tone thresholds for **(A)** left ear and **(B)** right ear. *p < 0.05 for group factor in repeated measures two-way ANOVA; ^¤^*p* < 0.05 for individual frequency comparison between groups in one-way ANOVA on the residuals.

Breachers exhibited poorer hearing thresholds in the right ear as analyzed from effect of group contribution to a two-way repeated measures ANOVA [*F*_(1,31)_ = 4.884, *p* = 0.035, Figure 2A]. This effect was not seen in identical analysis performed on threshold data from the left ear [*F*_(1,31)_ = 3.079, *p* = 0.089, Figure 2B]. *Post-hoc* analysis of the right ear residuals by ANOVA revealed the right ear group effect was most driven by hearing thresholds at 2,000 and 3,000 Hz [2,000 Hz: breacher residual = 0.450 ± 1.01, non-breacher residual = −0.610 ± 0.58, *F*_(1,31)_ = 12.222, *p* = 0.001; 3,000 Hz: breacher residual = 0.343 ± 1.11, non-breacher residual = −0.466 ± 0.59, *F*_(1,31)_ = 6.118, *p* = 0.019, Figure 2A]. The pattern of how hearing thresholds varied across the entire frequency spectrum was not different between breachers and non-breachers in either ear, as evidenced by a null interaction effect of frequency by group when analyzing the split-plot interaction of all variables in the aforementioned two-way repeated measures ANOVA (right ear *F* = 1.498, *p* = 0.213; left ear *F* = 1.716, *p* = 0.151).

No significant differences were observed for any vestibular or ocular motor outcome measures between breachers and non-breachers. Non-significant vestibular outcomes measures included VOR gain, phase and symmetry from rotational testing, caloric testing, and cervical VEMP P1-N1 amplitude and amplitude ratio. Overall mean smooth-pursuit eye movement parameters (velocity saccade percent, velocity gain asymmetry, and phase) and saccade eye movement parameters (accuracy grand mean, latency grand mean, and final accuracy grand mean) were not different between groups.

## Discussion

Recently, similar but more subtle and still deleterious symptoms of reduced hearing as well as cognitive deficits and increased audiologic complaints have been reported from individuals exposed to low-level overpressure environments (9). As such, it has become important to further elucidate, and even quantify, such adverse effects observed during low-level blast exposure, particularly as it relates to a growing concern for what constitutes safe occupational and militaristic operations when obligated to perform under controlled overpressure environments. For example, tinnitus is the most pervasive service-connected disability (16). In 2018, tinnitus was ranked first as the most prevalent disability for new Veterans Administration (VA) recipients (16). Similarly within our cohort, participants were more likely to report experiencing ringing in the ears than 11.2% of adults aged >18 in the general population (17).

Similar differences in subjective symptom reporting between breachers and non-breachers were observed in other circumstances as well. For example, the increased prevalence of self-reported photo- and phono-sensitivity among low-combat breachers vs. non-breachers was significantly higher. While it remains uncertain with respect to the degree that hearing differences cause breachers to report a greater sensitivity to noise, irritability might also have a concomitant impact on sensitivity to uncomfortable stimuli. However, it is important to consider that irritability might be associated with head injuries or military stress; a neuropsychological complement to this study will be discussed in future work.

The lack of difference between patterns of pure tone thresholds suggests that hearing loss occurs in a similar way among both groups, with some frequencies being more vulnerable than others. The significant group contribution to the model, at least in certain higher frequencies in the right ear, however, shows that breachers exhibit poorer hearing than non-breachers. This could be a result of a mostly right-handed sample: some weapons fired on the right side of the body of a right-handed individual may contribute, or hearing protection may more commonly be removed from the right ear of a right-handed individual when straining to hear or understand something. In breachers specifically, the increased hearing loss in the right ear might be an effect of training to face left prior to detonation.

These data support previous findings that identify subtle cognitive, otologic, and audiologic differences within a cohort of individuals subjected to controlled low-level sound overpressure. Such studies are often difficult to design and succeed in effective recruiting given the heterogenous nature of occupational experience, frequency of blast exposure, types of blast exposure, and any co-morbid historical or medical diagnoses such as childhood concussion. As such, the sample size for this study may limit the power of these results and should be interpreted with some caution. However, these findings are consistent with other recent work (18, 19), though it should be noted that breachers did not report headaches at a higher rate. In addition, self-reported responses are subjective and can be influenced by motivation to be perceived by others as ill or injured, or actually being perceived as such. Future blast and hearing research should take measures to include validated tinnitus scales and clinical emotion testing.

Finally, the lack of control for combat exposure was a shortcoming in characterizing long term exposure to blast. The recruitment of career breachers presents a robustly reliable blast exposure signal in the sample. However, the inherent nature of breaching involves entering structures and being within meters of combative individuals and combat environments. The Combat Exposure Checklist queries about sights and sounds experienced firsthand by the participant. By not controlling for combat exposure among the non-breachers in the sample, it is likely we recruited individuals who perform operations dozens or thousands of meters away from combat that can been seen and heard. Due to the presumed psychological impact of combat exposure on behavioral outcomes, it becomes challenging to attribute self-reported differences between the [otherwise well-matched] groups on breaching (and, by extension, blast exposure). Therefore, while worth mentioning the differences found in self-reported memory loss and difficulty concentrating, it may be just as likely these outcomes are associated with combat exposure as they are long term blast exposure. Future studies should include the Combat Exposure Checklist, or another scale like it, in order to better control for combat exposure or even to screen for participants. In a similar regard, until more is known about cumulative effects of exposure, studies should characterize and quantify all types of blast exposure so that even small arms exposure can be controlled for.

## Conclusions

When compared to non-breaching service and law enforcement members, breachers had more instances of deleterious hearing-related outcomes, irritability, and photo/phono-sensitivity while having no differences in vestibular or ocular motor responses. Breachers exhibited higher rates of pure tone hearing loss in one ear for frequencies commonly associated with noise exposure, as well as self-reported ringing in the ears, irritability, and sensitivity to light or noise. Vestibular and ocular motor outcome measures did not vary between groups. Breachers more often reported memory and concentration problems, but these outcomes were also associated with high combat exposure. Since the breachers sampled were characterized by higher combat exposure than the non-breachers, these outcomes require more research.

## Data Availability Statement

The dataset presented in this article is not yet available at the time of publication due to an ongoing study in progress. At the completion of the study, data will be uploaded to FITBIR. Requests to access the dataset can be directed to FITBIR-ops@mail.nih.gov at that time. In the meantime, requests can be sent to the corresponding authors.

## Ethics Statement

The studies involving human participants were reviewed and approved by Naval Medical Research Center; Walter Reed Army Institute of Research and the Central Nervous System IRB of the National Institutes of Health. The patients/participants provided their written informed consent to participate in this study.

## Author Contributions

CM and BJ prepared the initial draft. CM and BJ did the analyses. The authors that contributed in the writing and editing are: CM, BJ, CZ, KK, CB, JK, AY, ML, PW, KD, EP, AQ, BA, EW, JS, SA, and WC. All authors contributed to the article and approved the submitted version.

## Conflict of Interest

The authors declare that the research was conducted in the absence of any commercial or financial relationships that could be construed as a potential conflict of interest.
