# Supplementary material for: Hearing Loss and Irritability Reporting Without Vestibular Differences in Explosive Breaching Professionals
Source: Front Neurol. 2020 Dec 16;11:588377. doi: 10.3389/fneur.2020.588377 (PMC7772348; doi:10.3389/fneur.2020.588377)
Supplement: Supplementary file 1 [file Table_1.DOCX]

| Supplemental Table 1. Self-reported clinical interview responses | | | | | | | |
| --- | --- | --- | --- | --- | --- | --- | --- |
|  | | | % Reporting Yes | | | | |
|  |  |  | Breachers | | Non-breachers | | |
| 1 | Current diagnoses | | 25% | | 36% | | |
| 2 | Medical problems | | 5% | | 7% | | |
| 3 | Meningitis | | 0% | | 0% | | |
| 4 | Head injuries | | 70% | | 43% | | |
| 5 | Headaches | | 15% | | 14% | | |
| 6 | Tinnitus | | 65% | | 29% | | |
| 7 | Hearing loss | | 55% | | 29% | | |
| 8 | Ear discharge | | 5% | | 0% | | |
| 9 | Ear pain | | 5% | | 0% | | |
| 10 | Sinuses | | 30% | | 14% | | |
| 11 | Fainting/seizures | | 0% | | 0% | | |
| 12 | Vertigo | | 10% | | 14% | | |
| 13 | Balance | | 5% | | 7% | | |
| 14 | Coordination | | 5% | | 7% | | |
| 15 | Motion sickness | | 10% | | 14% | | |
| 16 | Gastrointestinal | | 5% | | 0% | | |
| 17 | Disturbing memories | | 5% | | 0% | | |
| 18 | Depression | | 15% | | 7% | | |
| 19 | Drug reactions | | 10% | | 0% | | |
| 20 | Back pain | | 60% | | 36% | | |
| 21 | Change of appetite | | 5% | | 0% | | |
| 22 | Avoiding stress | | 0% | | 7% | | |
| 23 | Memory | | 65% | | 29% | | |
| 24 | Decision-making | | 0% | | 7% | | |
| 25 | Concentration | | 55% | | 14% | | |
| 26 | Taste/smell | | 15% | | 0% | | |
| 27 | Going to sleep | | 50% | | 36% | | |
| 28 | Nightmares | | 15% | | 0% | | |
| 29 | Light-headed | | 10% | | 0% | | |
| 30 | Vision | | 20% | | 7% | | |
| 31 | Light/noise sensitive | | 30% | | 7% | | |
| 32 | Fatigue | | 20% | | 7% | | |
| 33 | Irritability | | 55% | | 14% | | |
| 34 | Disorientation | | 0% | | 0% | | |
| 35 | Stressful memories | | 0% | | 7% | | |
| 36 | Health concerns | | 15% | | 21% | | |
| 37 | Family neurological | | 50% | | 36% | | |
| 38 | Current prescriptions | | 40% | | 36% | | |
| 39 | Analgesic use | | 30% | | 43% | | |
| 40 | Tobacco use | | 20% | | 21% | | |
| 41 | Alcohol use | | 85% | | 64% | | |
| 45 | Sleep issues | | 60% | | 36% | | |
|  | | | | | | | |
|  | | Breachers | | | | Non-breachers | |
|  |  | Mean | | SD | | Mean | SD |
| 41 | Drinks per week | 4.0 | | 4.4 | | 2.3 | 3.5 |
| 42 | Exercise hours per week | 9.0 | | 6.1 | | 5.0 | 3.5 |
| 43 | Sleep hours per night | 6.1 | | 1.2 | | 6.2 | 1.4 |
| 44 | Optimal hours per night | 6.7 | | 1.2 | | 6.6 | 1.4 |
