# Supplementary material for: Hearing Loss and Irritability Reporting Without Vestibular Differences in Explosive Breaching Professionals
Source: Front Neurol. 2020 Dec 16;11:588377. doi: 10.3389/fneur.2020.588377 (PMC7772348; doi:10.3389/fneur.2020.588377)
Supplement: Supplementary file 2 [file Data_Sheet_1.DOCX]

Supplemental Appendix: Clinical Interview

All questions are yes/no unless otherwise indicated. Interviewers encouraged participants to elaborate on their responses when responses were “yes.”

Do you have a history of the following?

1. Currently diagnosed disease or injury
2. History of significant medical problem or injury
3. Meningitis
4. Head injury, concussion, or loss of consciousness
5. Troublesome headaches
6. Ringing in ears
7. Deafness or diminished hearing
8. Discharge (or drainage) from ears
9. Pain or fullness in the ear
10. Nose, sinus or throat trouble
11. Fainting attacks / Blackouts / Seizures
12. Dizziness / Vertigo
13. Balance problems
14. Coordination problems
15. Motion or travel sickness
16. Gastrointestinal problems / Nausea
17. Repeated disturbing memories, thoughts, or images
18. Depression, anxiety, or significant stress
19. Severe reaction to drug, injection
20. Backache, spinal injury, disc trouble, sciatica or lumbago
21. Change of appetite
22. Avoiding activities or situations that remind you of a stressful experience
23. Memory problems
24. Difficulty making decisions
25. Concentration problems
26. Change in taste or smell
27. Difficulty falling or staying asleep
28. Sleepwalking or frequent nightmares
29. Light-headedness
30. Eye trouble (blurry / shaky vision)
31. Sensitivity to light / noise
32. Fatigue / loss of energy
33. Irritability (easily annoyed)
34. Feeling disoriented
35. Physical reaction (heart pounding, sweating) when reminded of a stressful experience
36. Health concerns at present
37. Family neurological history
38. Current prescription medications
39. Over the counter analgesic use
40. Smoking/Tobacco use (# per day)
41. Alcohol use (drinks per week)
42. Exercise (hours per week)
43. Sleep actually getting (hours per night)
44. Sleep needed to feel rested (hours per night)
45. Sleep issues
46. Head injuries (# per age range: under 7, 7-15, 16-34, and 35+ years of age)
